# Gestational Diabetes Mellitus in Pregnancy Increased Erythropoietin Level Affecting Differentiation Potency of Haematopoietic Stem Cell of Umbilical Cord Blood

**DOI:** 10.3389/fmed.2021.727179

**Published:** 2021-08-19

**Authors:** Mohd Razif Mohd Idris, Fazlina Nordin, Zaleha Abdullah Mahdy, S. Fadilah Abd Wahid

**Affiliations:** ^1^Cell Therapy Centre, Universiti Kebangsaan Malaysia Medical Centre, Kuala Lumpur, Malaysia; ^2^Centre for Tissue Engineering and Regenerative Medicine, Universiti Kebangsaan Malaysia Medical Centre, Kuala Lumpur, Malaysia; ^3^Department of Obstetrics and Gynaecology, Universiti Kebangsaan Malaysia Medical Centre, Kuala Lumpur, Malaysia

**Keywords:** umbilical cord blood, gestational diabetes, hematopoietic stem cell, colony forming unit, erythropoietin

## Abstract

**Background:** The *in utero* environment has many factors that can support cell differentiation. Cytokines, chemokines and growth factors play big roles in haematopoietic mechanisms. Some diseases like gestational diabetes mellitus (GDM) might affect the environment and haematopoietic stem cell (HSC) quality. The aim of this study is to investigate the adverse effects of GDM on umbilical cord blood (UCB) HSC in terms of differentiation potency including the UCB parameters used for banking and transplantation purposes.

**Methods:** UCB-HSC was collected from 42 GDM and 38 normal pregnancies. UCB-HSC was isolated and further enriched using immuno-magnetic separation beads (MACS). The UCB-HSC were cultured in methylcellulose media to investigate the differentiation potency. The level of erythropoietin (EPO) and insulin in the UCB plasma was measured using enzyme linked immunoassay (ELISA) technique.

**Result:** The UCB parameters; volume, total nucleated count (TNC) and total CD34+ cells were significantly reduced in the GDM group compared to the control group. The number of HSC progenitors' colonies were significantly reduced in the GDM group except for progenitor BFU-E, which was significantly increased (GDM = 94.19 ± 6.21, Control = 73.61 ± 2.73, *p* = 0.010). This data was associated with higher EPO level in GDM group. However, the insulin level in the GDM group was comparable to the Control group.

**Conclusion:** Our results suggest that the changes in the *in utero* environment due to abnormalities during pregnancy such as GDM might affect the differentiation potency of UCB-HSC. These findings can be considered as an additional parameter for the inclusion and exclusion criteria for UCB banking, particularly for mothers with GDM.

## Introduction

UCB has been used as an alternative source of haematopoietic stem cells (HSCs) for transplantation. Over 30,000 UCB transplants have been performed worldwide for the treatment of various diseases. Although the volume of UCB is small at around 100 mL per unit, it is capable of growing faster and enabling it to regenerate the entire of HSC population compared to bone marrow (1). UCB offers the advantages of easier acquisition, lack of risk for donors (mothers), less risk of transmitted infection, immediate availability and less stringent criteria for the human leucocyte antigen (HLA) matching and low incidence of graft vs. host disease (2). However, insufficient number of HSCs in one unit UCB causes the slowdown in haematopoietic recovery, especially in adult and child patients compared to infant or small children patients (3). The successful outcome of transplantation is closely correlated to the nucleated cell count (NCC) and the number of CD34^+^ cells (4, 5).

The ideal number of NCC for transplantation is 1 × 10^7^ to 3 ×10^7^ cells/kg of recipient weight, whereby the higher the dose of NCC, the shorter the engraftment period (1, 6). Meanwhile for CD34^+^ cells, the best dose for transplantation is >1.7 × 10^5^ cells/kg that are disparate in <2 human leukocyte antigens (HLA). One finding showed that only 22% of patients were able to survive more than 1 year after receiving NCC <3.7 x 10^7^ cells/kg, while 41% of patients receiving at least 3.7 × 10^7^ cells/kg had more than 1 year of survival (2). While one study found that patients receiving CD34^+^ cells <1.7 × 10^5^ cells/kg and NC 1 × 10^7^ cells/kg had a higher mortality rate (4). Based on these findings, most UCB banks have set policies for selecting high quality UCB units for storage purposes (7).

To date, very few studies have been conducted to examine the effects of gestational diabetes mellitus (GDM) on the quality of UCB-HSC. GDM is defined as glucose intolerance with onset or first recognition during pregnancy. Previous study on a single centre experience at Hospital Universiti Kebangsaan Malaysia (UKM), reported an overall poor outcome of GDM pregnancy with high level of HbA1c, but the effect on the quality of UCB-HSC was not further investigated (8). Data from developed countries suggest that the prevalence of GDM is increasing, being almost 10% of pregnancies and probably reflecting the global obesity epidemic. In our centre, the prevalence can be as high as 24.9% (9). While considering longer term outcomes for the baby, evidence is gradually mounting that GDM adds an intrauterine environmental risk factor (10). GDM may affect placental haemodynamics with abnormal umbilical artery blood flow which can alter the *in utero* environment such as EPO and insulin (11) resulting in foetal hypoxia.

EPO is a hormone that plays a role in regulating foetal erythropoiesis (12). A pathology has been detected in the process of erythropoiesis in infants of diabetic mothers, attributable to abnormalities in the metabolic system and intra-uterine hypoxia (12, 13).

Meanwhile, the high levels of insulin in the UCB plasma could interfere with foetal growth and subsequently alter the process of angiogenesis and vascularization that could affect the quality of UCB-HSC (11). These two cytokines, EPO and insulin, may provide a good microenvironment for cell growth and haematopoiesis (14), thus producing good quality HSC. High EPO levels always correlated with diabetic pregnancies and hypoxia, whilst higher insulin might impair the match between supply and demand from the placenta. This study aims to investigate whether GDM might influence the quality and *in vitro* differentiation of UCB-HSC and its correlation with EPO and insulin levels.

## Methods

### Subjects

This prospective case-control study was conducted on 42 pregnancies with GDM, and 38 healthy pregnancies, which were screened and recruited at 20–24 weeks of gestation and on monthly follow-up and admitted for delivery at Universiti Kebangsaan Malaysia Medical Centre (UKMMC). The study was approved by the Research Ethics Committee of UKMMC. GDM was defined according to the American College of Obstetricians and Gynaecologists guidelines with abnormal glucose values at fasting and 2 h post-prandial (75 g OGTT test) (15). Healthy subjects (the Control group) consisted of pregnant women of similar age, parity and gestational age without any serious disease, neither preeclampsia nor GDM. All subjects must have blood screening for hepatitis B, hepatitis C, cytomegalovirus, syphilis and human immunodeficiency virus 1-2 as per hospital practise. Screening for genetic diseases, pregestational diabetes mellitus, chronic hypertension, autoimmune diseases, renal or liver impairment and haematological disorders as per medical history. Screening for multiple pregnancies and foetal anomalies or infection were performed using the detailed scan ultrasound. Additional File 1 shows the summary of the experimental design of the present study.

### Umbilical Cord Blood Collection

UCB collections were performed by trained staff nurses in accordance with hospital protocol during delivery. It was performed before placental dismissal in all patients to avoid interfering with the delivery of the baby while still preserving the sterility of the UCB. Briefly, the cord was clamped and cut after delivery of the baby. A four to eight inches' length of the cord was cleaned with alcohol and betadine. A 16-gauge needle from a standard cord blood collection bag set containing 22 mL of citrate phosphate dextrose (CPD) anticoagulant was inserted into the umbilical vein and cord blood was allowed to flow freely into a 157 mL collection bag. The needle was removed when blood flow stopped. An identical collection method was used for the control group. Blood samples were stored at 4°C immediately after collection before being collected by the laboratory personnel. Samples were processed within 24 h after collection.

### Mononuclear Cell Collection and CD34+ Cells Selection

The UCB mononuclear cells (MNCs) were extracted through density centrifugation using Lymphoprep solution (Stem Cell Technologies, USA). Briefly, the MNC layer was aspirated into new tubes and suspended with Phosphate-buffered saline (PBS) (1:3 ratios) and centrifuged at 1,000 rpm for 10 min. The pellet was washed twice with PBS and resuspended in 300 uL of Iscove's Modified Dulbeccos's Medium (IMDM). CD34+ cell selection was performed according to the manufacturer's protocol (MACS CD34 MicroBead Kit Human). Briefly, 100 uL of blocking reagent and 100 uL of CD34^+^ microbeads solution was added, followed by 30 min' incubation at 2–8°C. The microbeads-conjugated cells were separated using a magnetic column following cell counting by haemocytometers.

### Colony Forming Unit Assay

CFU assay was performed to observe the differentiation potency of the umbilical cord blood-haematopoietic stem cell (UCB-HSC) enriched CD34+ cells and to quantify the numbers of each differentiated haematopoietic progenitor cells. The assay was performed using a Methocult™ Kit (Stem cell Technologies, USA). Briefly, a total of 5 × 10^3^ of enriched CD34+ cells were seeded in 35 mm petri dish with differentiation media, and further incubated at 37°C for 14 days. The HSC differentiated colonies; *colony-forming unit granulocyte, erythrocyte, monocyte, megakaryocyte* (CFU-GEMM), *colony-forming unit granulocyte, monocyte* (CFU-GM), *burst-forming unit erythrocyte* (BFU-E), *colony-forming unit monocyte* (CFU-M), and *colony-forming unit granulocyte* (CFU-G) were counted under an inverted microscope. Experiments were done in duplicate for each sample. Additional File 2 shows the morphology of UCB-HSC progenitor cell differentiation from a control subject as reference.

### Insulin Assay

The plasma insulin level was determined using Quantikine® ELISA Human Insulin (R&D Systems™ a bio-techne brand, USA) according to the manufacturer's protocol. Briefly, the plasma taken during MNC density centrifugation, control and standard samples were added to the coated wells to allow antigen-antibody binding. The plate was incubated at room temperature, and washed four times. The biotinylated antibody was added to each well. The excess detection antibody was removed, and an enzyme (Streptavidin-HRP) was added and incubated to complete the four-member sandwich binds (*antibody-antigen-detection antibody-enzyme*). After the second incubation, the plate was washed four times to remove all unbound enzymes, and the final solution (stabiliser substrate solution) was added to produce the colour of the substrate-enzyme linked product. The intensity of the coloured product (optical density, OD) was compared to the concentration of human insulin, which can be determined using an ELISA plate reader at 450 nm wavelength. The concentration of human insulin in tested samples can be obtained by comparing the OD of the samples to the standard. Overall incubation time was 4 h in total with 30 min' hands-on time. Each sample was run in duplicate.

### Erythropoietin Assay

The plasma EPO level was determined using pre-coated Human EPO ELISA Kit (Elabscience, Biotechnology Inc. USA) according to manufacturer's protocol. Briefly, standards and samples (plasma) were added to a microplate and combined with the specific antibody. After the first incubation, the plate was washed four times and the biotinylated antibody was added, following Streptavidin-HRP in each well. The plate was further incubated, washed and the enzyme-substrate reaction was terminated by adding the stop solution. The OD was measured using an ELISA plate reader at 450 nm wavelength. The OD is proportionate to the concentration of human EPO. The concentration of human EPO in tested samples can be obtained by comparing the OD of the samples to the standard. Overall incubation time was 3 h in total with 30 min' hands-on time. Each sample was run in duplicates.

### Clinical Data and Statistical Analysis

The clinical data for each subject were obtained from the patient's medical records. The statistical analyses were performed using SPSS 25.0 software (RRID:SCR_002865). Normality test was performed using Shapiro-Wilk test. Continuous data were analysed using independent *t*-test with equal variances assumed. Furthermore, categorical data were analysed using Fisher's Exact Test. The correlation between UCB volume and UCB parameters was calculated using Spearman's rank-order test. Data were shown in mean ± SD and significance is denoted with a *p*-value of <0.05.

## Results

### Clinical Data Analysis

Overall, the mean age of women with GDM in this study was 30.81 years (range between 28–34 years old (Table 1). Body mass index (BMI) was significantly higher in women with GDM compared to the Control group (*p* < *0.0005*). The average gestational age at delivery was 37.89 in the GDM group, which was significantly lower compared to the Control group (*p* = *0.031*) (Table 1). The rate of caesarean section in the GDM group relatively high compared to the Control group (*p* = *0.028*). T (Table 1).

**Table 1:** A comparison of maternal & foetal characteristics in GDM and control groups.

**Parameter**	**GDM (***n*** = 42)**	**Control (***n*** = 38)**	***P*** **-value**
Mother's age	30.81 ± 0.58	29.89 ± 0.54	0.534¥
Body mass index (BMI)	26.7 ± 0.4	22.8 ± 0.5	**<0.0005** ¥
Pregnancy duration (week)	37.89 ± 0.22	38.55 ± 0.19	**0.031** ¥
Leucocyte count (× 10^9^/L)	11.04 ± 0.53	11.06 ± 0.43	0.978¥
*Gravidity			
1–3	35 (83.33%)	36 (94.74%)	0.159‡
4–6	7 (16.66%)	2 (5.26%)	
Systolic pressure (mmHg)	120.74 ± 1.57	120.21 ± 1.81	0.825¥
Diastolic pressure (mmHg)	70.49 ± 1.49	69.38 ± 1.47	0.459¥
*Delivery method			
SVD	32 (47.05%)	36 (52.95%)	**0.028** ‡
LUSCS	10 (83.33%)	2 (16.67%)	
Baby weight (kg)	3.03 ± 0.07	3.08 ± 0.06	0.916¥
Placenta weight (g)	582.70 ± 16.81	600.00 ± 15.71	0.747¥

¥ *Independent sample t-test (Data shown in mean ± SD)*.

‡ *Fisher's Exact test*.

*p value is significant < 0.05*.

* *Shown as frequency*.

*SVD, spontaneous vaginal delivery; LUSCS, lower uterine segment caesarean section. Bold values meaning of significant value*.

### UCB Volume and UCB Parameters Analysis

The correlation between UCB volume and parameters such as nucleated cell count (NCC), total nucleated cell (TNC), CD34^+^ cell count and total CD34^+^ cell was further analysed (Table 2). Our data showed all UCB parameters were significantly decreased in the GDM group compared to the Control group (*p* < 0*.0005*) (Table 2). The TNC and CD34+ cell counts in the GDM group were half lower than the Control group (Table 2).

**Table 2:** A comparisons of UCB parameters in GDM and control groups.

**Parameter**	**GDM (***n*** = 42)**	**Control (***n*** = 38)**	***P*** **-value**
Volume (mL)	80.36 ± 1.88	102.16 ± 2.43	**<0.0005**
NCC (× 10^6^ cell/mL)	4.74 ± 0.30	9.03 ± 0.46	**<0.0005**
TNC (× 10^8^ cell)	3.87 ± 0.29	9.36 ± 0.62	**<0.0005**
CD34^+^ count (cell/μL)	61.18 ± 6.64	125.60 ± 9.75	**<0.0005**
Total CD34^+^ Count (× 10^6^ cell)	4.88 ± 0.55	13.07 ± 1.11	**<0.0005**

*Independent sample t-test (Data shown in mean ± SD)*.

*p-value is significant < 0.05*.

*NCC, nucleated cell count; TNC, total nucleated count*.

*Bold values meaning of significant value*.

### EPO Analysis

**Figure 1:**
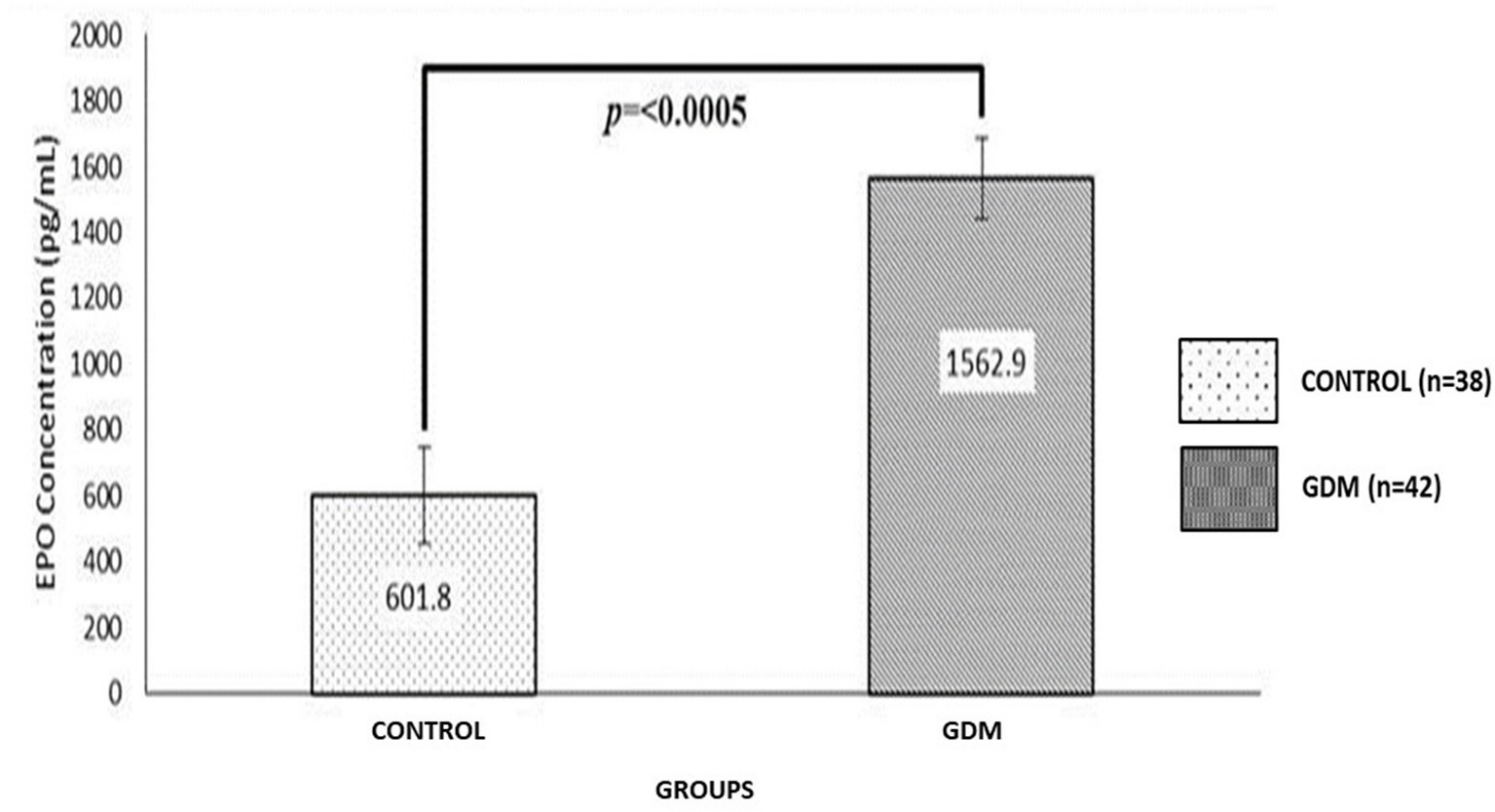
Comparison of EPO UCB concentration (pgmol/mL) between GDM and Control groups. The EPO level was significantly higher in GDM as compared to the Control group (*p* = < 0.0005).

The average EPO concentration for the GDM and Control groups were 1,562.9 pg/mL, and 601.8 pg/mL, respectively (Figure 1). The EPO level was significantly higher in the GDM group compared to the Control group (*p* < 0*.0005*) (Figure 1). Overall, there was no correlation between UCB parameters with the EPO level in both GDM and the Control group, except for the reduced TNC counts, which was significantly correlated with a higher EPO level in the GDM group (*p* = 0.033) (Table 3).

**Table 3:** A correlation between UCB parameters and EPO level in GDM and Control groups.

**Group**	**EPO**	**Volume (ml)**	**NCC (× 10^**6**^)**	**TNC (× 10^**8**^)**	**CD34^**+**^ (cell/μL)**	**Total CD34^**+**^ (× 10^**6**^)**
Control (*n* = 38)	Nilai *p*	0.859	0.896	0.956	0.215	0.202
	Nilai r	−0.030	−0.022	0.009	−0.206	−0.212
GDM (*n* = 42)	Nilai *p*	0.087	0.243	**0.033**	0.072	0.612
	Nilai r	0.268	0.184	0.329	0.281	0.080

*p-values based on statistic Spearman's rank order statistical analysis*.

*p-value is significant < 0.05*.

*NCC, nucleated cell count; TNC, total nucleated count*.

*Bold values meaning of significant value*.

### Insulin Analysis

**Figure 2:**
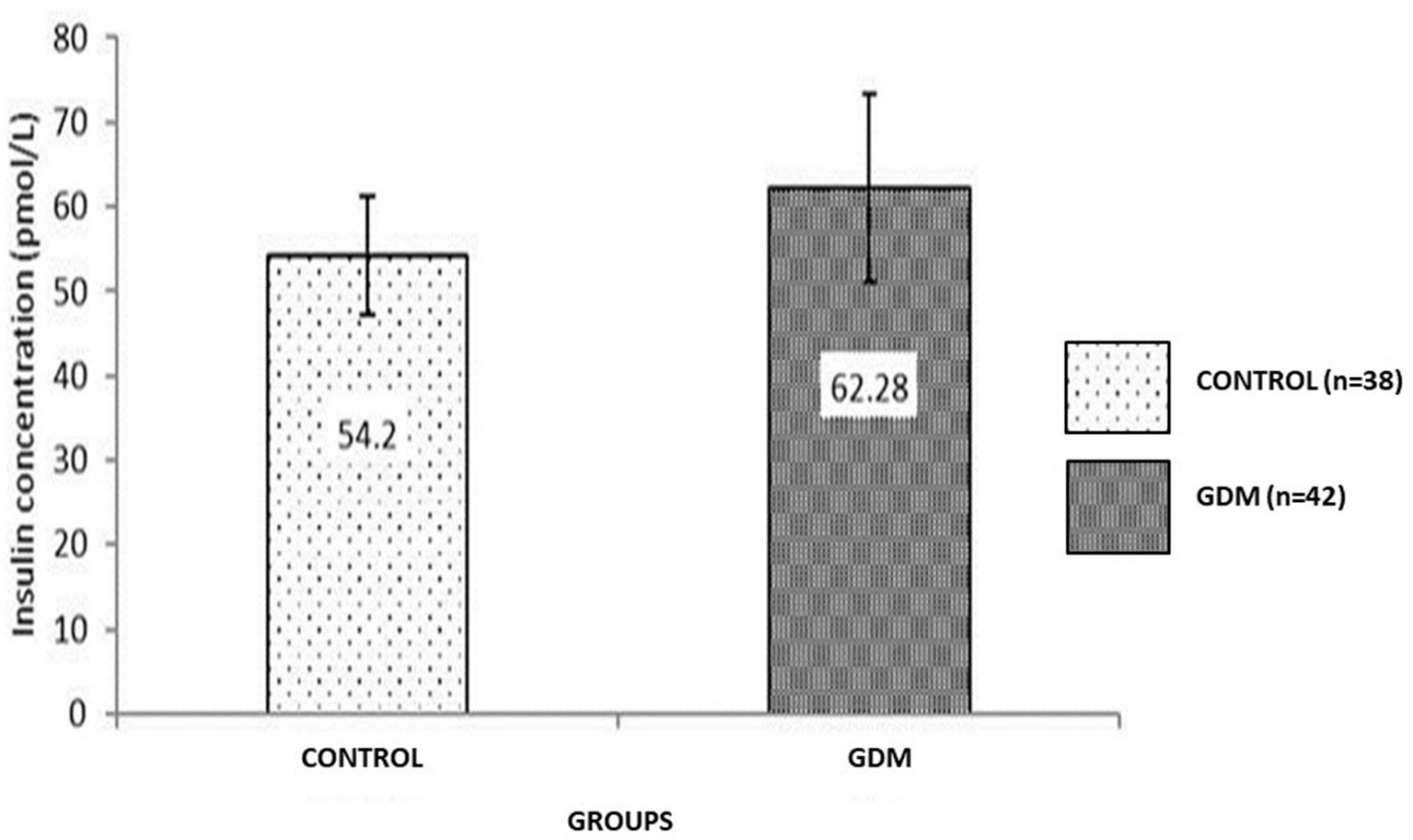
Comparison of insulin UCB (pmol/L) concentration between GDM and Control groups. The insulin level in the GDM group is comparable with the Control group. Data shown is mean ± SEM.

The average insulin concentrations for the GDM and Control groups were 62.28 pmol/L and 54.2 pmol/L, respectively (Figure 2). There was no significant difference between the two groups studied. Overall, there was no significant correlation between UCB parameters and insulin level in the GDM and Control groups (Table 4).

**Table 4:** A correlation between UCB parameters and insulin level in GDM and control groups.

**Group**	**Insulin**	**Volume (ml)**	**NCC (× 10^**6**^)**	**TNC (× 10^**8**^)**	**CD34^**+**^ (cell/μL)**	**Total CD34^**+**^ (× 10^**6**^)**
Control (*n* = 38)	Nilai *p*	0.558	0.465	0.674	0.959	0.646
	Nilai r	−0.098	0.122	0.071	0.009	−0.077
GDM (*n* = 42)	Nilai *p*	0.730	0.456	0.658	0.411	0.908
	Nilai r	−0.055	0.118	0.070	−0.130	−0.018

*p-values based on statistic Spearman's rank order statistical analysis*.

*p-value is significant < 0.05*.

*NCC, nucleated cell count; TNC, total nucleated count*.

### The Haematopoietic Differentiation of CD34+ Enriched Cell by CFU Assay

We observed the colony forming units of all types of progenitors at 14 days' post-incubation. The CFU-GEMM (*p* < 0.0005), CFU-GM (*p* < 0.0005), CFU-G (*p* = 0.001), and CFU-M (*p* < 0.0005) colonies were significantly reduced in the GDM group compared to the Control group (Table 5). Interestingly, this study showed a high number of BFU-E colonies forming in the GDM group (94.19 ± 6.21) compared to the Control group (73.61 ± 2.73) (*p* = *0.01*) (Table 5).

**Table 5:** A comparison of progenitors counts in GDM and Control groups.

**Types of progenitor**	**GDM (***n*** = 42)**	**Control (***n*** = 38)**	***P*** **-value**
CFU-GEMM	65.98 ± 2.98	87.45 ± 1.46	**<0.0005**
CFU-GM	49.50 ± 3.06	70.92 ± 1.93	**<0.0005**
BFU-E	94.19 ± 6.21	73.61 ± 2.73	**0.010**
CFU-G	70.55 ± 4.00	89.21 ± 1.46	**0.001**
CFU-M	62.79 ± 3.93	81.39 ± 1.76	**<0.0005**

*Independent sample t-test (Data shown in mean ± SD)*.

*p-value is significant < 0.05*.

*CFU-GEMM, colony forming unit granulocyte erythrocyte monocyte megakaryocyte; CFU-GM, colony forming unit granulocyte monocyte; BFU-E, burst forming unit erythroid; CFU-G, colony forming unit granulocyte; CFU-M, colony forming unit megakaryocyte*.

*Bold values meaning of significant value*.

## Discussion

### Statement of Principle Findings

This study has demonstrated that GDM in pregnancy affected the UCB volume, TNC and total CD34+ cell counts. Furthermore, the CFU assay has confirmed the adverse effect of GDM in terms of the ability of UCB-HSC to differentiate into progenitor colonies. The GDM group has shown a significantly reduced number of CFU colonies except for high BFU-E suggesting an active erythropoiesis process due to abnormal placental blood flow and hypoxia. These data also demonstrated a high EPO level in the GDM group. The EPO level was significantly higher in GDM, however the insulin level was comparable to that of the Control group.

### Findings in Context of Existing Research

#### Gestational Age Is Shorter in the Group of GDM Than in the Control Group

Gestational age factor showed a significant difference between the group of GDM mothers compared to the Control group (*p* = *0.031*) with an average week at 37.89 for the GDM group. This early birth may be due to the risk of the disease itself or iatrogenic syndrome which requires the patient to give birth earlier (16). These data are in line with the relatively high percentage of caesarean delivery methods (83.33%) in the GDM group.

#### UCB Volume Decreased in GDM Pregnancy Probably Due to Placental Microvascular Pathology

The UCB volume in the GDM group was significantly lower than the Control group (*p* < 0.0005) (Table 2). This difference was as expected based on the pathophysiology of diabetes mellitus. It has been reported that the production of prostacyclin, a blood vessel development agent, is reduced in DM patients, thus leading to vasoconstriction of *utero*-placental blood vessels (17). Therefore, a smaller volume of UCB will be obtained. For UCB banking, the UCB volume is a crucial criterion in order to enable further processing and retrieval, as this will determine the number of CD34+ cell needed for downstream treatment.

#### NCC and TNC Counts Were Reduced in GDM Pregnancy and Did Not Meet the International Cell Transplant Standards for Processing and Cryopreservation

The NCC and TNC values in the GDM group were half than the Control group. According to the standards of UCB banks, the minimum value of TNC for processing and cryopreservation is 4–6 × 10^8^ cells (18, 19). However, the average value of TNC in the GDM group was 3.87 × 10^8^ cells (Table 2), which may be considered for processing and cryopreservation. TNC count is an important parameter for prediction of the survival and mortality outcomes following HSC transplantation. The TNC transplant required a standard TNC count of 1–3 × 10^7^ cell/kg weight of the receiver. A higher TNC count will give better transplant outcomes including reduced mortality (18).

#### Total CD34^+^ Cell Count Reduced in GDM Group and Only Sufficient for Child Recipients

The GDM group had significantly lower total CD34+ cell counts compared to the Control group. The number of CD34^+^ cells is an important parameter in determining the success of transplantation in terms of post-transplant survival and mortality. A study that was conducted in patients who received a higher CD34^+^ cell count (>1.7 × 10^5^ CD34^+^/kg) showed a reduced mortality rate (4). Our data showed a mean total of 4.88 × 10^6^ CD34+ cells in the GDM group (Table 2), suggesting that children aged between 5–11 years (with <28 kg weight) would be suitable recipients.

#### The Insulin Concentration of UCB in the GDM Group Is Comparable to the Control Group and Does Not Affect HSC Quality

Early and intensive treatment provided by the clinical team at UKMMC to pregnancy mothers' with GDM likely contributed to a controlled insulin level. This was done through a dietary plans and insulin control. The UCB insulin assessment of the GDM group is very useful in monitoring the level of foetal exposure to glucose *in utero*. The foetus exposed to hyper-insulinemia has the potential to develop illnesses such as diabetes, abnormal glucose tolerance and metabolic complications later on (20). However, the association between the level of insulin and HSC quality is not well-studied. It was also reported that angiogenesis and vascularization changes may affect the quality of HSC (11).

#### GDM Group Shows Reduced Number of Differentiated Haematopoietic Progenitor Colonies as Compared to Control Group

These findings are in line with previous studies that reported pregnant mothers with preeclampsia (PE) (21). However, there is as yet no such published research on GDM patients. The generation of HSC in placenta and cord during pregnancy is quite complex and not well-understood. Gekas et al. suggested that the placental microenvironment is quite rich with cytokines and growth factors, properties that support the expansion of HSC (22). In theory, GDM could alter placental microenvironment properties (22), thus suggesting a reduced progenitors count in the GDM group. In addition, the higher number of progenitor cells in the Control group may be due to the decent quality of CD34^+^ cells. The CD34^+^ cell is a haematopoietic cell that undergoes haematopoiesis in clonogenic assay (23). The high number of colonies in the Control group indicates that these unobstructed CD34^+^ cells are able to differentiate and sprout.

#### The Number of BFU-E Colonies Is Higher in the GDM Group Compared to the Control Group

BFU-E is a progenitor cell colony formed from over 200 erythroblasts that undergo erythropoiesis. This finding is similar to the previous study on UCB of premature infants (24). However, the study of Wisgrill et al. (24) showed a high number of CD34^+^ cells in premature UCB, and suggested stress in these premature infants causing instability of both cytokine and chemokine levels. This phenomenon gives impact to the haematopoiesis process (24). However, in this study, the CD34^+^ cell count in the GDM group was lower than the Control group, thus suggesting that the high number of colonies of BFU-E is likely due to an active erythropoiesis process. Erythropoiesis is the process mediated by EPO which produces red blood cells. In addition, high oxygen demand by foetal hyper-insulinaemia syndrome also affects the process of aerobic metabolism that causes changes in the erythropoiesis process. In this study, the EPO assay was performed to further investigate the correlation between EPO level and HSC quality, predominantly with the high number of BFU-E colonies in the GDM group.

#### The High Number of BFU-E Colonies Is Probably Due to an Increased Level of EPO in the GDM Group

Generally, the quality of HSC is translated as the HSC's ability to differentiate and form various haematopoietic colonies observed through the CFU assay. A high EPO level in the GDM group may also explain the high number of BFU-E colonies. According to literature studies, most cases of GDM show increased foetal oxidative stress, which is thought to play a role in maternal and foetal complications of diabetic pregnancies (25). Oxidative stress is also related to the degree of increased EPO production and hence polycythaemia in the infant. This leads to an increase in EPO levels in UCB following activation of erythropoiesis processes (26) and occurrence of polycythaemia in the newborn (27). This suggests that an increased number of BFU-E colony in the GDM group corresponds to an increased EPO level, probably due to hypoxia affecting the placenta.

Preliminary findings from this study suggest that GDM influenced the quality of UCB HSC. These results would be useful for physicians and mothers in relation to decision on UCB banking. In addition, studies on cytokines such as osteopontin and other growth factors and correlation with neonatal outcomes (i.e., birth weight, apgar score, Hb concentration, haematocrit) should be considered in order to better comprehend their effect on the quality of UCB-HSC and their association with placental development.

## Limitations

We are aware of some limitations of our study. First, the study was performed on a limited sample size. Another limitation is that the study was performed in a single hospital, possibly leading to poor representation of the general population.

## Future Studies

A larger sample in future may help confirm our findings in relation to EPO and CFU assays. We suggest extension of such a study to other hospitals in future as a multicenter research. Also important to look into neonatal outcomes to correlate with findings.

## Data Availability Statement

The original contributions presented in the study are included in the article/Supplementary Material, further inquiries can be directed to the corresponding author/s.

## Ethics Statement

The studies involving human participants were reviewed and approved by Research Ethics Committee of Universiti Kebangsaan Malaysia (UKM) under the Centre of Research & Instrumentation UKM with the reference number (UKM 1.5.3.5/244/FRGS/2/2013/SKK01/UKM/03/2). The patients/participants provided their written informed consent to participate in this study.

## Author Contributions

MM conducted the study. FN, ZM, and SA contributed to the design of the study. MM and FN wrote this manuscript and involved in the collection of the data and analysis. All authors contributed to the article and approved the submitted version.

## Conflict of Interest

The authors declare that the research was conducted in the absence of any commercial or financial relationships that could be construed as a potential conflict of interest.

## Publisher's Note

All claims expressed in this article are solely those of the authors and do not necessarily represent those of their affiliated organizations, or those of the publisher, the editors and the reviewers. Any product that may be evaluated in this article, or claim that may be made by its manufacturer, is not guaranteed or endorsed by the publisher.
