# Supplementary material for: Gestational Diabetes Mellitus in Pregnancy Increased Erythropoietin Level Affecting Differentiation Potency of Haematopoietic Stem Cell of Umbilical Cord Blood
Source: Front Med (Lausanne). 2021 Aug 19;8:727179. doi: 10.3389/fmed.2021.727179 (PMC8416672; doi:10.3389/fmed.2021.727179)
Supplement: Supplementary file 1 [file Presentation_1.PPTX]

## Slide 1
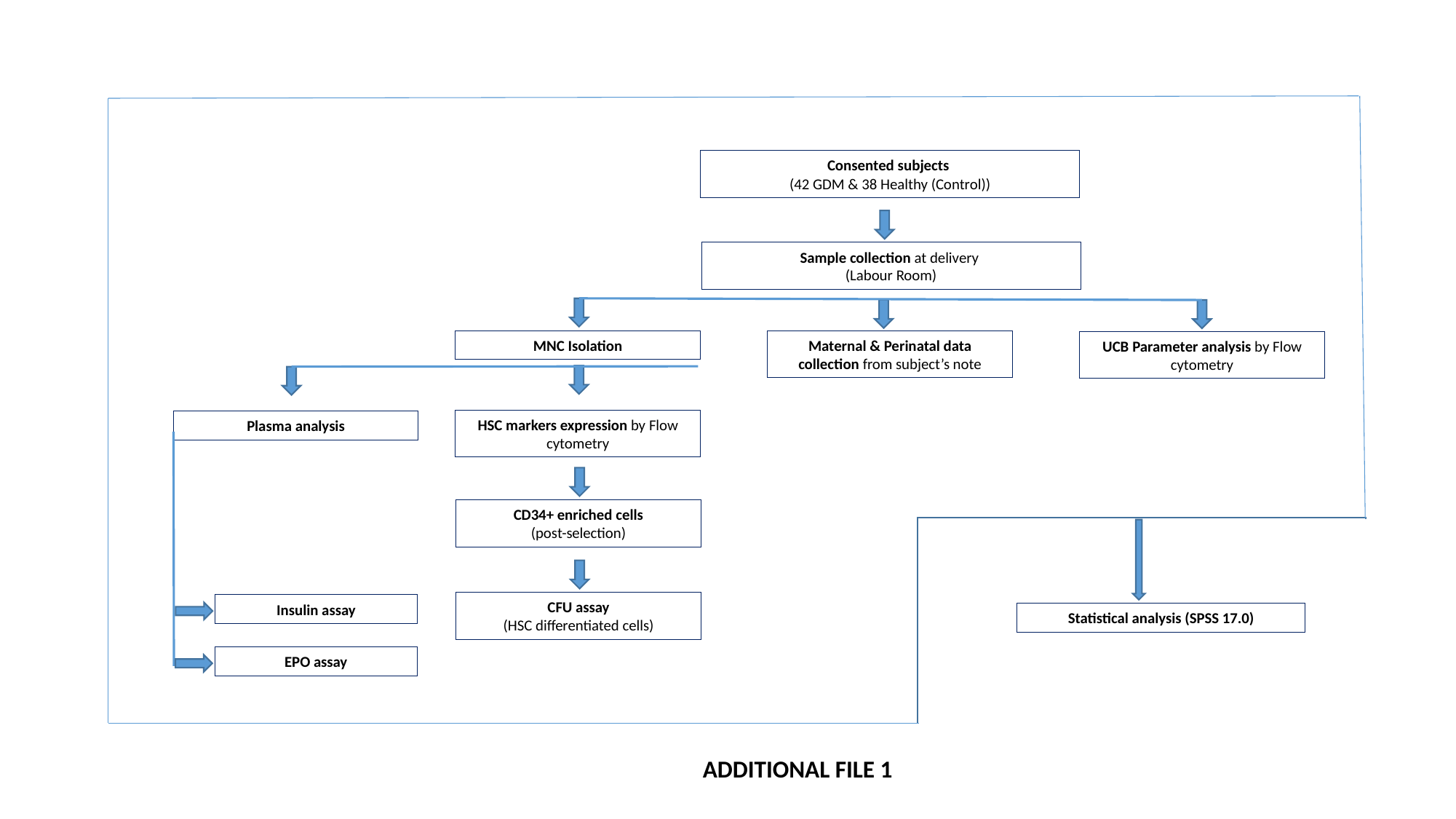

Consented subjects
(42 GDM & 38 Healthy (Control))
Sample collection at delivery
(Labour Room)
MNC Isolation
Maternal & Perinatal data collection from subject’s note
UCB Parameter analysis by Flow cytometry
HSC markers expression by Flow cytometry
Plasma analysis
CD34+ enriched cells
(post-selection)
CFU assay
(HSC differentiated cells)
Insulin assay
Statistical analysis (SPSS 17.0)
EPO assay
ADDITIONAL FILE 1
