# Supplementary figures and images for: Gestational Diabetes Mellitus in Pregnancy Increased Erythropoietin Level Affecting Differentiation Potency of Haematopoietic Stem Cell of Umbilical Cord Blood
Source: Front Med (Lausanne). 2021 Aug 19;8:727179. doi: 10.3389/fmed.2021.727179 (PMC8416672; doi:10.3389/fmed.2021.727179)

## Slide 1
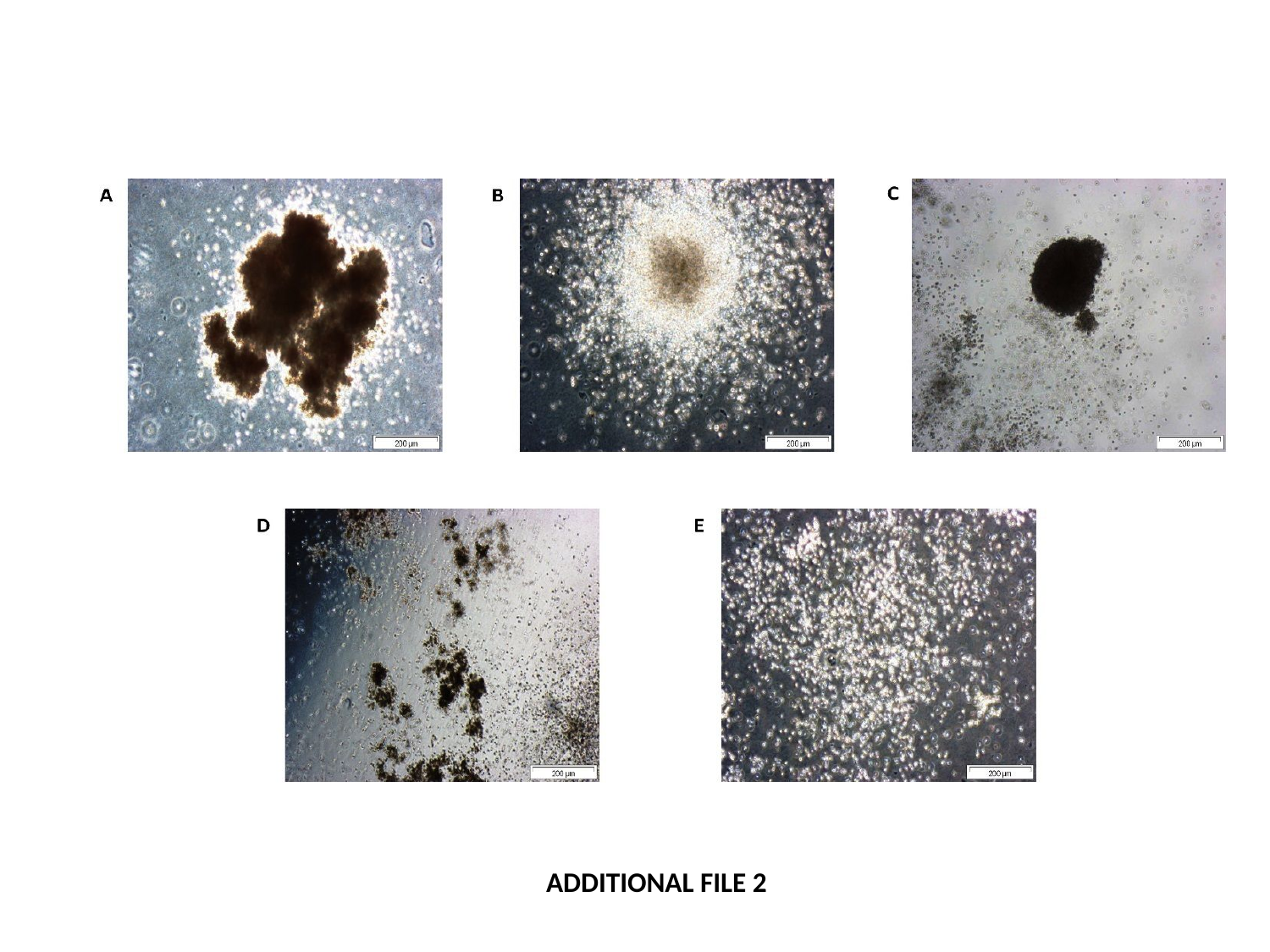

ADDITIONAL FILE 2

Supplement: Supplementary file 2 [file Presentation_2.PPTX]
